# Budesonide Multimatrix Is Efficacious for Mesalamine-refractory, Mild to Moderate Ulcerative Colitis: A Randomised, Placebo-controlled Trial

**DOI:** 10.1093/ecco-jcc/jjx032

**Published:** 2017-03-04

**Authors:** David T. Rubin, Russell D. Cohen, William J. Sandborn, Gary R. Lichtenstein, Jeffrey Axler, Robert H. Riddell, Cindy Zhu, Andrew C. Barrett, Enoch Bortey, William P. Forbes

**Affiliations:** a Inflammatory Bowel Disease Center, University of Chicago Medicine, Chicago, IL, USA; b Division of Gastroenterology, University of California [UC] San Diego and UC San Diego Health System, San Diego, CA, USA; c Division of Gastroenterology, Perelman School of Medicine of the University of Pennsylvania, Philadelphia, PA, USA; d Toronto Digestive Disease Associates, Toronto, ON, Canada; e Department of Pathology and Laboratory Medicine, Mt Sinai Hospital, Toronto, ON, Canada; f Salix Pharmaceuticals, Raleigh, NC, USA

**Keywords:** Ulcerative colitis, inflammatory bowel disease, inflammation, clinical trials

## Abstract

**Background and Aims:**

Safety and efficacy of budesonide multimatrix, an oral extended-release second-generation corticosteroid designed for targeted delivery throughout the colon, were examined for induction of remission in patients with mild to moderate ulcerative colitis refractory to baseline mesalamine therapy.

**Methods:**

A randomised, double-blind, placebo-controlled, multicentre trial evaluated efficacy and safety of budesonide multimatrix for induction of remission [ulcerative colitis disease activity index score ≥ 4 and ≤ 10] in 510 adults randomised to once-daily oral budesonide multimatrix 9 mg or placebo for 8 weeks. Patients continued baseline treatment with oral mesalamine ≥ 2.4 g/day.

**Results:**

Combined clinical and endoscopic remission at Week 8 was achieved by 13.0% and 7.5% of patients receiving budesonide multimatrix [*n* = 230] or placebo [*n* = 228], respectively, in the modified intention-to-treat population [*p* = 0.049]. Clinical remission [ulcerative colitis disease activity index rectal bleeding and stool frequency subscale scores of 0] was similar in both groups [*p* = 0.70]. More patients receiving budesonide multimatrix vs placebo achieved endoscopic remission [ulcerative colitis disease activity index mucosal appearance subscale score of 0; 20.0% vs 12.3%; *p* = 0.02] and histological healing [27.0% vs 17.5%; *p* = 0.02]. Adverse event rates were similar [budesonide multimatrix, 31.8%; placebo, 27.1%]. Mean morning cortisol concentrations decreased at Weeks 2, 4, and 8 with budesonide multimatrix but remained within the normal range.

**Conclusion:**

Budesonide multimatrix was safe and efficacious for inducing clinical and endoscopic remission for mild to moderate ulcerative colitis refractory to oral mesalamine therapy.

## 1. Introduction

Ulcerative colitis [UC] is a chronic inflammatory bowel disease that affects the colonic mucosa, extending proximally from the rectum.^1^ Clinical symptoms of UC include rectal bleeding, diarrhoea, urgency, tenesmus, and abdominal pain.^1^ Current treatment guidelines recommend that patients with active, mild to moderate UC receive either rectal mesalamine for more distal forms of UC, or oral mesalamine for more extensive forms of the disease.^2^^,^^3^ Systemic corticosteroids are usually prescribed after patients fail to respond to mesalamine therapy,^2^^,^^3^ but are associated with potentially serious adverse events [AEs] including infections, ophthalmological complications, and cushingoid features.^2^ Budesonide is a second-generation corticosteroid that undergoes extensive first-pass hepatic metabolism due to its low systemic bioavailability [~ 10%].^4^^,^^5^ Budesonide multimatrix [MMX] extended-release tablets were developed to pass through the stomach intact and release active drug throughout the length of the colon.^6^^,^^7^

Results of two phase 3, randomised, double-blind, placebo-controlled studies [colonic release budesonide (CORE) I^8^ and CORE II^9^] of patients with active, mild to moderate UC demonstrated that budesonide MMX 9 mg induced combined clinical and endoscopic remission in a significantly greater percentage of patients compared with placebo [CORE I, 17.9% vs 7.4%, respectively, *p* = 0.0143; CORE II, 17.4% vs 4.5%, respectively, *p* = 0.005] and had a favourable safety profile comparable with placebo. Concomitant use of other UC therapies was not permitted in these studies.^8^^,^^9^ Potential glucocorticoid-related adverse effects [eg, moon face, fluid retention, sleep changes] occurred infrequently in both groups, and mean morning plasma cortisol concentrations decreased with budesonide MMX but remained within normal levels during both studies.

A small phase 2 study consisting of a 4-week randomised, double-blind, placebo-controlled phase followed by a 4-week open-label extension phase, demonstrated that clinical improvement or remission was achieved by 47.1% and 33.3% of patients who received budesonide MMX 9 mg or placebo, respectively, for treatment of UC, despite concomitant oral mesalamine use.^10^ Budesonide MMX 9 mg had a favourable safety profile; mean cortisol concentrations decreased after 4 weeks with budesonide MMX 9 mg, but remained within normal range for the duration of the study.

These findings suggest that budesonide MMX may be included in treatment algorithms after mesalamine has failed, but before systemic corticosteroid use.^11^ The current study evaluated budesonide MMX 9 mg administered once daily for 8 weeks for the induction of remission of mild to moderate UC not adequately controlled by stable, oral mesalamine therapy.

## 2. Materials and Methods

### 2.1. Patients

Patients aged 18 to 75 years with active UC [ulcerative colitis disease activity index [UCDAI] mucosal appearance subscale score ≥ 1] by flexible sigmoidoscopy at screening, as well as mild to moderate UC [baseline UCDAI score ≥ 4 and ≤ 10, mucosal appearance subscore ≥ 1, and physician’s rating of disease activity score of 1 or 2, despite receiving oral mesalamine ≥ 2.4 g/day (or equivalent) for ≥ 6 weeks before randomisation] were included. Previously randomised patients without histological evidence of active UC [as determined by central histopathological reading] continued the study but were not included in the primary efficacy analysis population.

Exclusion criteria included: evidence of limited distal proctitis [extending from the anal verge up to 15 cm above the pectineal line]; Crohn’s disease or indeterminate colitis; microbiologically confirmed infectious colitis or a history of infectious colitis within 30 days of screening; history of pancolitis [disease extending to the hepatic flexure or beyond] for ≥ 8 years or left-sided colitis [disease distal to the splenic flexure] for ≥ 15 years without surveillance colonoscopy for dysplasia/colorectal cancer screening within the past year; or liver cirrhosis, hepatic or renal disease or insufficiency, or ≥ 2.5 times the upper limit of normal [ULN] for alanine aminotransferase, aspartate aminotransferase, gamma glutamyl transferase, or ≥ 2 times ULN for creatinine. Patients could not have received previous treatment with: budesonide MMX; oral corticosteroids, including other formulations of budesonide, during the previous 4 weeks; any rectal mesalamine or corticosteroid formulation during the previous 2 weeks; immunosuppressive agents during the previous 8 weeks; or biologic therapies during the previous 3 months. Systemic or rectal steroids, any mesalamine other than the existing oral mesalamine at the same dose a patient was receiving at study initiation, anti-tumour necrosis factor-α agents and other biologics, and immunosuppressants were prohibited.

The protocol was approved by institutional review boards and ethics committees. All patients provided written informed consent. All authors had full access to the study data and reviewed and approved the final manuscript.

### 2.2. Study design

This phase 3, multicentre, randomised, double-blind, placebo-controlled study [NCT01532648] was conducted in the USA, Canada, and Europe between December 2011 and December 2012. Patients were randomised 1:1 to receive budesonide MMX 9 mg or placebo once daily after breakfast for 8 weeks in an outpatient setting. Patients were assigned to treatment groups via an interactive voice response system [IVRS] using computer-generated randomisation and stratification by study centre. Patients continued treatment with the same preparation and dosage of oral mesalamine [or equivalent] reported at study entry. Minimum required doses were ≥ 2.4 g/day for mesalamine, ≥ 4.0 g/day for sulphasalazine, ≥ 2.0 g/day for olsalazine, or ≥ 6.75 g/day for balsalazide. The study consisted of a 2-week screening phase, an 8-week treatment phase, and a 4-week posttreatment phase [Supplementary Figure 1, available as Supplementary data at *ECCO-JCC* online].

### 2.3. Assessments

The primary efficacy endpoint was the percentage of patients achieving combined clinical and endoscopic remission [total UCDAI score, according to Sutherland,^12^ of ≤ 1, with subscale scores of 0 for rectal bleeding, stool frequency, and mucosal appearance] at Week 8. Secondary efficacy outcomes included the percentage of patients achieving clinical remission [UCDAI subscale scores of 0 for rectal bleeding and stool frequency] and the percentage of patients achieving endoscopic remission [UCDAI subscale score of 0 for mucosal appearance] at Week 8. An exploratory endpoint included the percentage of patients achieving histological healing [histological activity grade of 0] at Week 8. For this endpoint, three mucosal biopsies were taken from the most severely affected colonic region[s] during endoscopy procedures performed at screening and Week 8; biopsies were analysed and scored by a blinded, independent histopathologist at a central laboratory using the Geboes system.^13^ Additional exploratory endpoints included the percentage of patients with clinical improvement [≥ 3-point improvement from baseline in UCDAI score and a rectal bleeding score ≤ 1] at Week 8, and serum C-reactive protein [CRP] and faecal calprotectin concentrations at Week 8. Quality of life [QOL] was evaluated at Weeks 2, 4, and 8 using the inflammatory bowel disease quality of life [IBD-QOL] instrument, a 32-item questionnaire comprising bowel function, emotional function, systemic symptoms, and social function dimensions, with higher scores indicating improved QOL. Safety assessments included AE monitoring, potential predefined glucocorticoid-related adverse effects [ie, moon face, striae rubrae, flushing, fluid retention, mood changes, sleep changes, insomnia, acne, hirsutism], clinical laboratory tests [including morning cortisol concentrations and adrenocorticotrophic hormone (ACTH) challenge tests], physical examinations, and vital sign measurements. A normal response to the ACTH challenge test was defined as an increase from baseline in plasma cortisol concentration > 7 µg/dl, or a plasma cortisol concentration > 18 µg/dl 30 min post-ACTH challenge.^14^

### 2.4. Statistical analyses

The intention-to-treat [ITT] population included all randomised patients who received ≥ 1 dose of study drug and had active UC at study entry as a cause of symptoms [based on photographic mucosal evidence]; the safety population included all patients who received ≥ 1 dose of study drug. The modified ITT [mITT] population included all patients in the ITT population with histological evidence of active UC and no evidence of enteric infection. Based on an assumption of remission rates of 15% for placebo and 27% for budesonide MMX, a total of 250 patients per group would be expected to provide 90% power to detect a significant difference between the two groups with a two-sided α = 0.05. Compliance with budesonide MMX was determined by daily self-report of study drug administration through the IVRS, as well as by subtracting the amount of drug returned at Weeks 4 and 8 from the amount of drug dispensed. The Wilcoxon rank sum test was used for tests of continuous variables, and the chi-square method was used for tests of binomial proportions, unless the expected cell frequency was less than five for more than one cell in a two x two table; in that case, Fisher’s exact test was used. Missing data for patients in the mITT population were handled using a worst-case imputation scheme, where patients with missing data were considered nonresponders; clinical improvement, IBD-QOL, and serum CRP data were analysed using the last observation carried forward method.

## 3. Results

### 3.1. Patient disposition and demographics

**Figure 1.**
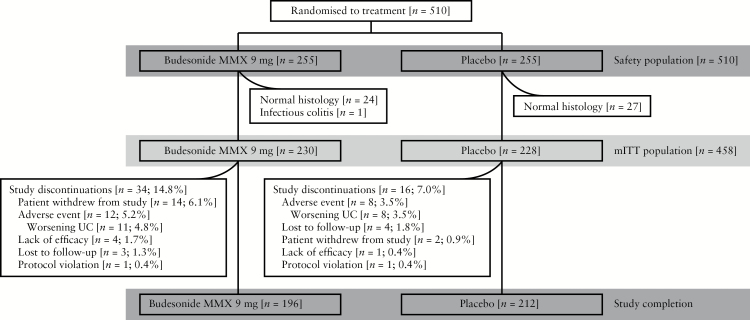
Patient disposition. mITT, modified intention-to-treat; MMX, multimatrix; UC, ulcerative colitis.

Of the 510 randomised patients who received ≥ 1 dose of study drug [ITT population], 52 were excluded from efficacy analyses due to normal histology based on central laboratory reading [*n* = 51] or infectious colitis [*n* = 1]; the mITT population included 230 and 228 patients receiving budesonide MMX or placebo, respectively. For the mITT population, 85.2% and 93.0% of patients receiving budesonide MMX or placebo, respectively, completed the study [Figure 1]. Demographic and baseline disease characteristics were generally comparable between treatment groups [Table 1]. The majority of patients received concomitant treatment with mesalamine at doses ≥ 2.4 g/day but < 4.8 g/day.

**Table 1.** Demographic and baseline characteristics, modified intention-to-treat population.

Characteristic	Budesonide MMX 9 mg *n* = 230	Placebo *n* = 228
Age, years, mean [SD]	44.5 [14.1]	44.6 [13.7]
Sex, male, *n* [%]	121 [52.6]	127 [55.7]
Race, White, *n* [%]	219 [95.2]	210 [92.1]
BMI, kg/m^2^, mean [SD]	25.7 [5.2]	25.6 [5.0]
Duration of disease, months, mean [SD]	80.4 [91.0]	78.9 [90.5]
Duration of current flare, *n* [%]		
≤ 4 weeks	43 [18.7]	36 [15.8]
5–12 weeks	87 [37.8]	94 [41.2]
> 12 weeks	90 [39.1]	94 [41.2]
Missing	10 [4.3]	4 [1.8]
Extent of disease, *n* [%]		
Proctosigmoiditis	94 [40.9]	85 [37.3]
Left-sided UC	84 [36.5]	94 [41.2]
Extensive colitis	13 [5.7]	16 [7.0]
Pancolitis	39 [17.0]	33 [14.5]
Severity of current flare, *n* [%]		
Mild	42 [18.3]	47 [20.6]
Moderate	188 [81.7]	181 [79.4]
Baseline UCDAI total score, mean [SD]	6.5^a^ [1.5]	6.6 [1.6]
Baseline UCDAI rectal bleeding subscore, *n* [%]		
0	29 [12.6]	34 [14.9]
1	128 [55.7]	111 [48.7]
2	69 [30.0]	77 [33.8]
3	3 [1.3]	6 [2.6]
Baseline UCDAI stool frequency subscore, *n* [%]		
0	28 [12.2]	25 [11.0]
1	85 [37.0]	85 [37.3]
2	69 [30.0]	75 [32.9]
3	47 [20.4]	43 [18.9]
Baseline UCDAI mucosal appearance subscore, *n* [%]		
0	0	0
1	40 [17.4]	43 [18.9]
2	161 [70.0]	155 [68.0]
3	29 [12.6]	30 [13.2]
Baseline UCDAI physician’s rating of disease activity subscore, *n* [%]		
0	0	0
1	45 [19.6]	49 [21.5]
2	185 [80.4]	179 [78.5]
3	0	0
Total daily dose of background mesalamine equivalent, *n* [%]		
< 2.4 g	33 [14.3]	42 [18.4]
≥ 2.4 g to < 4.8 g	170 [73.9]	158 [69.3]
≥ 4.8 g	27 [11.7]	28 [12.3]
Mean dose, g [SD]	3.1 [1.4]	3.0 [1.2]
Prior biologic therapy use, *n* [%]^b^		
Adalimumab	2 [0.8]	1 [0.4]
Golimumab	0	1 [0.4]
Infliximab	10 [3.9]	9 [3.5]

BMI, body mass index; MMX, multimatrix; SD, standard deviation; UC, ulcerative colitis; UCDAI, ulcerative colitis disease activity index.

^a^Data missing for 1 patient.

^b^Data presented for safety population; budesonide MMX [*n* = 255] and placebo [*n* = 255].

### 3.2. Efficacy

**Figure 2.**
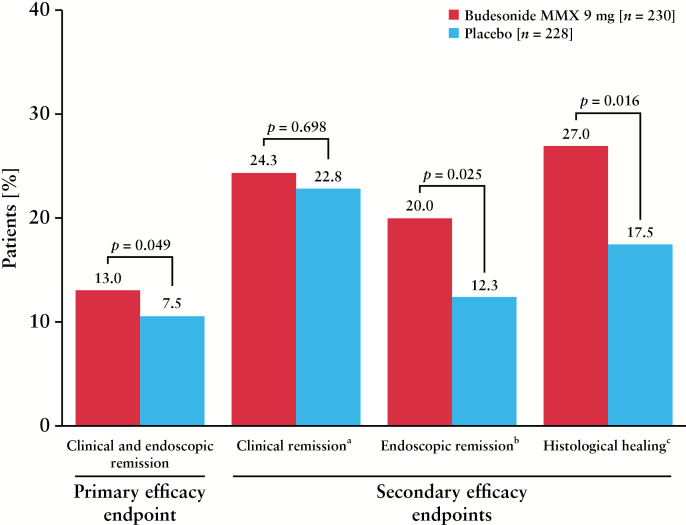
Patients achieving primary and secondary efficacy outcome measures at Week 8. MMX, multimatrix. ^a^Clinical remission defined as rectal bleeding and stool frequency subscores = 0. ^b^Endoscopic remission defined as mucosal appearance subscore = 0. ^c^Histological healing defined as histological activity grade = 0 [normal mucosa], by central reading.

A significantly greater percentage of patients receiving budesonide MMX 9 mg achieved combined clinical and endoscopic remission after 8 weeks [primary efficacy endpoint] compared with placebo [13% vs 7.5%, respectively; *p* = 0.049; worst-case analysis; Figure 2]. The UCDAI mucosal appearance score was the primary driver for this effect, with 20.0% and 12.3% of patients in the budesonide MMX or placebo groups, respectively, achieving a mucosal appearance subscore of 0 [*p* = 0.02].

A comparable percentage of patients receiving budesonide MMX 9 mg and placebo achieved clinical remission [ie, UCDAI rectal bleeding and stool frequency subscale scores of 0; 24.3% vs 22.8%, respectively; *p* = 0.70]. A greater percentage of patients receiving budesonide MMX 9 mg achieved endoscopic remission compared with placebo after 8 weeks [20.0% vs 12.3%, respectively; *p* = 0.025; Figure 2]. The percentage of patients with a UCDAI rectal bleeding subscore of 0 was comparable between groups [budesonide MMX, 48.7%, vs placebo, 47.8%]; a similar percentage of patients in each group achieved a UCDAI stool frequency score of 0 [budesonide MMX, 34.3%, vs placebo, 31.1%]. Histological healing was achieved by a significantly greater percentage of patients receiving budesonide MMX 9 mg compared with placebo [27% vs 17.5%, respectively; *p* = 0.016]. No significant differences were noted between budesonide MMX [47.0%] and placebo [39.0%; *p* = 0.09] for the exploratory endpoint of percentage of patients with clinical improvement at Week 8. Serum CRP concentrations decreased from baseline to Week 8 with only placebo, whereas faecal calprotectin concentrations decreased from baseline in both groups; for both biomarkers, the change from baseline was comparable between groups [Supplementary Table 1, available as Supplementary data at *ECCO-JCC* online]. Total and subscale scores of the IBD-QOL instrument were improved from baseline with both budesonide MMX and placebo by Week 2, and maintained through Week 8 [Supplementary Table 2, available as Supplementary data at *ECCO-JCC* online].

### 3.3. Safety

Budesonide MMX 9 mg was well tolerated when administered in combination with oral mesalamine, as the majority of AEs [28.7%] with budesonide MMX were mild to moderate in intensity. Overall, 31.8% and 27.1% of patients receiving budesonide MMX or placebo, respectively, reported AEs, with the most common AE in both groups being UC [Table 2]. Serious AEs occurred in a small percentage of patients receiving budesonide MMX [UC (2.4%), pancreatitis (0.4%), bronchitis (0.4%), anaemia (0.4%), hypokalaemia (0.4%)] or receiving placebo [UC (0.4%), type 2 diabetes mellitus (0.4%)]. Study discontinuation due to AEs occurred in 4.7% and 3.5% of patients receiving budesonide MMX or placebo, respectively. Potential glucocorticoid-related AEs occurred in 9.0% and 5.9% of patients receiving budesonide MMX or placebo, respectively, with moon face [3.1% vs 2.0%, respectively], sleep changes [2.0% vs 1.6%], fluid retention [1.6% vs 2.0%], and mood changes [0.4% vs 2.0%] reported by at least 2% of patients in either treatment group.

**Table 2.** Summary of adverse events, safety population.

Adverse event, *n* [%]	Budesonide MMX *n* = 255	Placebo *n* = 255
Any AEs	81 [31.8]	69 [27.1]
Drug-related AEs	31 [12.2]	15 [5.9]
Discontinuations due to AE	12 [4.7]	9 [3.5]
Serious AEs^a^	10 [3.9]	2 [0.8]
Drug-related serious AEs^b^	2 [0.8]	0
AE intensity		
Mild	44 [17.3]	41 [16.1]
Moderate	29 [11.4]	26 [10.2]
Severe	8 [3.1]	2 [0.8]
Most common AEs^c^		
UC	15 [5.9]	10 [3.9]
Decreased blood cortisol levels	10 [3.9]	0
Acne	3 [1.2]	5 [2.0]
Serious AEs		
UC	6 [2.4]	1 [0.4]
Acute pancreatitis	1 [0.4]	0
Bronchitis	1 [0.4]	0
Anaemia	1 [0.4]	0
Hypokalaemia	1 [0.4]	0
Type 2 diabetes mellitus	0	1 [0.4]

AE, adverse event; MMX, multimatrix; UC, ulcerative colitis.

^a^Serious AEs reported in the budesonide MMX group: UC [*n* = 6], anaemia [*n* = 1], acute pancreatitis [*n* = 1], bronchitis [*n* = 1], hypokalaemia [*n* = 1]. Serious AEs reported in the placebo group: UC [*n* = 1], type 2 diabetes mellitus [*n* = 1].

^b^Drug-related serious AEs included UC and acute pancreatitis.

^c^Common AEs included those reported in ≥ 2% of patients in either group.

**Figure 3.**
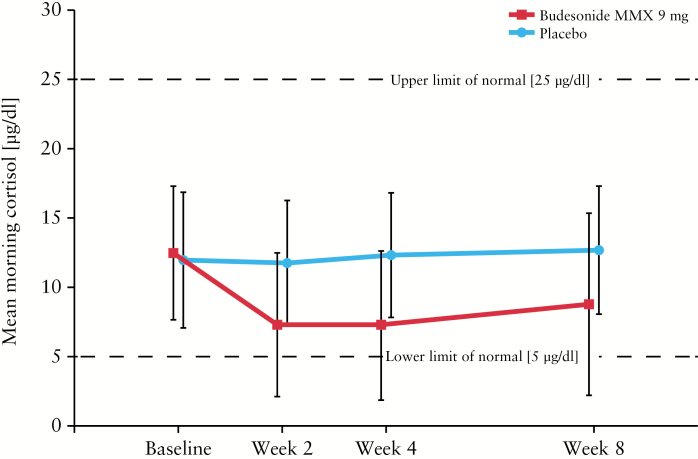
Mean morning plasma cortisol concentrations. MMX, multimatrix.

Mean morning plasma cortisol concentrations were within normal levels in both treatment groups at baseline, Week 2, Week 4, and Week 8 [Figure 3; Supplementary Table 3, available as Supplementary data at *ECCO-JCC* online]. Mean cortisol concentrations after ACTH stimulation were comparable at baseline in patients receiving budesonide MMX and placebo [22.3 µg/dl and 21.7 µg/dl, respectively]. However, mean cortisol concentrations after ACTH stimulation were below normal with budesonide MMX after 8 weeks [15.6 µg/dl]; patients receiving placebo had cortisol concentrations comparable with baseline after 8 weeks [22.3 µg/dl]. The majority of patients maintained normal total concentrations and had normal response to ACTH challenge during the study [Table 3].

**Table 3.** Total cortisol concentrations and normal response to ACTH challenge

Parameter, *n*/*N*^a^ [%]	Budesonide MMX 9 mg *n* = 255	Placebo *n* = 255
Total cortisol > 5 µg/dl^b^		
Baseline	249/255 [97.6]	241/255 [94.5]
Week 2	146/244 [59.8]	237/249 [95.2]
Week 4	144/241 [59.8]	233/242 [96.3]
Week 8	150/225 [66.7]	231/236 [97.9]
Normal response to ACTH challenge^c^		
Baseline	222/248 [89.5]	223/254 [87.8]
Week 8	119/224 [53.1]	202/236 [85.6]

ACTH, adrenocorticotrophic hormone; MMX, multimatrix.

^a^Denominator *N* is the number of patients with a value at each given week during the study.

^b^Lower limit of normal.

^c^Defined as increase from baseline in plasma cortisol concentration > 7 µg/dl, or plasma cortisol concentration > 18 µg/dl 30 min after ACTH challenge.

## 4. Discussion

The results of this randomised, double-blind, placebo-controlled study demonstrated the efficacy, safety, and tolerability of once-daily, oral budesonide MMX 9 mg for the induction of remission of patients with mild to moderate UC unresponsive to oral mesalamine monotherapy. All patients in this study continued treatment with a stable dose of oral mesalamine in addition to the study drug, in contrast with the CORE I and CORE II studies, which prohibited concomitant oral mesalamine.^8^^,^^9^ However, more than 50% of patients in the CORE I and CORE II studies reported previous use of mesalamine, with 54.5% of patients in CORE I reporting mesal amine exposure within 14 days of randomisation.

The percentage of patients receiving budesonide MMX or placebo who achieved combined clinical and endoscopic remission was comparable between this study and the CORE I and CORE II studies.^8^^,^^9^ The results of the primary endpoint of the current study were primarily driven by the UCDAI mucosal appearance score, with a significantly greater percentage of patients achieving endoscopic remission with budesonide MMX after 8 weeks compared with placebo. The percentage of patients achieving a stool frequency score of 0 was comparable between groups; however, budesonide MMX may not have a large effect on this component of the UCDAI, as 20% to 43% of patients with active UC may have formed stools.^15^ Clinical remission [ie, rectal bleeding and stool frequency subscale scores of 0], a subjective secondary outcome measure based on patient-reported outcomes, was not statistically significant for budesonide MMX compared with placebo. However, the objective secondary endpoints of endoscopic remission and histological healing were significant for budesonide MMX vs placebo in this study. Histological and endoscopic scores have been shown to be positively correlated,^16^ but it is unclear why the percentage of patients with histological healing was greater than the percentage of patients with combined clinical and endoscopic remission, as many patients with UC in clinical remission also have histological evidence of inflammation.^16^ Rectal bleeding and stool frequency are components of the definition of clinical remission that may not be caused by inflammation, which may account for the finding of histological healing in this study. Serum CRP concentrations, a biomarker of inflammation, were unchanged from baseline with budesonide MMX and decreased with placebo at Week 8; it is unclear why decreased serum CRP concentrations were only observed in patients receiving placebo, but it may be due to poor assay sensitivity.^17^ The decrease from baseline in faecal calprotectin concentrations was comparable between groups at Week 8. Of note, these biomarkers are currently not recommended for assessing therapeutic response.^17^

A limitation of this study was the stringent definition of remission, which may have limited the number of patients who achieved the primary and secondary efficacy outcomes. However, more stringent definitions of remission help decrease the incidence of false-positives in a study.^18^ Combined clinical and endoscopic remission is considered an appropriate therapeutic endpoint in clinical studies of UC.^19^ Clinical remission is an important endpoint for patients, as the rectal bleeding and stool frequency components of the Mayo score have performed well as indicators of patient perception of clinical response to therapy.^18^ However, although sigmoidoscopy and colonoscopy are invasive procedures not viewed favourably by most patients,^18^ mucosal healing, shown by the absence of mucosal ulceration and erosions, has been suggested as the ultimate goal of treatment in patients with UC.^20^ Treatment with corticosteroids or oral mesalamine has been associated with an increased likelihood for mucosal healing within 1 year in patients with UC.^21^ Histological healing has not been historically evaluated as a therapeutic endpoint in clinical studies of patients with UC and, when evaluated, has been limited by lack of validation and standardisation of how histological endpoints are scored, reported, and defined.^19^^,^^22^ Thus, the percentage of patients who achieved the primary and secondary efficacy outcomes in the current study was most likely limited by the rigorous definition of remission, so direct comparisons with previously published studies of mild to moderate UC may not apply.

This study did not confirm endoscopic scoring using indepen dent, central readers, which may have affected endoscopic outcomes. Feagan *et al*.^23^ previously noted that site readers overestimated outcomes only with placebo treatment in a study of mesalamine, resulting in greater treatment differences vs active drug; however, the statistical significance between treatments remained unchanged. Further, use of flexible sigmoidoscopy, rather than colonoscopy, may not be as informative in patients with more extensive UC. Another limitation of this study was the absence of safety and efficacy data beyond 8 weeks. To date, data regarding the safety and efficacy of budesonide MMX for maintenance of remission have not been published. Long-term follow-up data regarding duration of remission and relief of clinical symptoms in patients who achieved clinical and/or endoscopic remission in this study are not available. Additional clinical studies are warranted to examine these unanswered questions.

The safety profile of budesonide MMX was favourable and comparable with that of other studies examining similar doses of budesonide MMX in patients with active, mild to moderate UC.^8^^,^^9^ Thus, concomitant treatment with oral mesalamine had no apparent effect on the incidence of AEs with budesonide MMX, although some serious AEs occurred in patients receiving budesonide MMX and not in those receiving placebo [ie, pancreatitis, bronchitis, anaemia, hypokalaemia: all 0.4% each]. The incidence of potential glucocorticoid-related effects was low and comparable with reports from previous clinical studies of budesonide MMX.^8^^,^^9^ Importantly, mean morning plasma cortisol concentrations remained within normal range during the 8-week study. In conclusion, once-daily oral budesonide MMX is a well-tolerated, efficacious therapeutic option for induction of combined endoscopic and clinical remission in mild to moderate UC, including in patients with UC not adequately controlled with oral mesalamine therapy alone.

## Funding

This work was supported by Santarus, Inc., previously a wholly owned subsidiary of Salix Pharmaceuticals [Raleigh, NC]. The trial was designed and managed by Santarus, Inc. [San Diego, CA]. The data were housed and analysed by Santarus, Inc., and Salix, in collaboration with study investigators. The authors had full access to the data.

## Conflict of Interest

DTR has received research grants and served as a consultant for Salix Pharmaceuticals and Santarus, Inc. [previously a wholly owned subsidiary of Salix]. RDC has served as a consultant and advisory board participant for Salix and Santarus, Inc. WJS has received consulting fees from Salix and Santarus, Inc. GRL has received research grants and/or served as a consultant for Salix and Santarus, Inc. JA has nothing to disclose. RHR has served as a consultant for Salix for this project. CZ, ACB, EB, and WPF were employees of Salix at the time the analyses were conducted.

## Author Contributions

All authors participated in drafting of the manuscript or critical revision of the manuscript for important intellectual content, and provided approval of the final submitted version. DTR was involved with: conception and design of the study; generation, collection, assembly, analysis, and/or interpretation of data; drafting and revision of the manuscript; and approval of the final manuscript for submission. RDC was involved with: conception and design of the study; generation, collection, assembly, analysis, and/or interpretation of data; drafting and revision of the manuscript; and approval of the final manuscript for submission. WJS was involved with: conception and design of the study; analysis and/or interpretation of data; drafting and revision of the manuscript; and approval of the final manuscript for submission. GRL was involved with: conception and design of the study; generation, collection, assembly, analysis and/or interpretation of data; drafting and revision of the manuscript; and approval of the final manuscript for submission. JA was involved with: interpretation of data analyses; drafting and critical revision of the manuscript; and approval of the final manuscript for submission. RHR was involved with: interpretation of data analyses; drafting and critical revision of the manuscript; and approval of the final manuscript for submission. CZ was involved with: interpretation of data analyses; drafting and critical revision of the manuscript; and approval of the final manuscript for submission. ACB was involved with: interpretation of data analyses; drafting and critical revision of the manuscript; and approval of the final manuscript for submission. EB was involved with: interpretation of data analyses; drafting and critical revision of the manuscript; and approval of the final manuscript for submission. WPF was involved with: interpretation of data analyses; drafting and critical revision of the manuscript; and approval of the final manuscript for submission.

## Supplementary Data

Supplementary data are available at *ECCO-JCC* online.

## Supplementary Material

Supplementary Material
