# Corrigendum: Budesonide Multimatrix Is Efficacious for Mesalamine-refractory, Mild to Moderate Ulcerative Colitis: A Randomised, Placebo-controlled Trial

**DOI:** 10.1093/ecco-jcc/jjx052

**Published:** 2017-04-27

**Authors:** David T Rubin, Russell D Cohen, William J Sandborn, Gary R Lichtenstein, Jeffrey Axler, Robert H Riddell, Cindy Zhu, Andrew C Barrett, Enoch Bortey, William P Forbes

**Affiliations:** 1 Inflammatory Bowel Disease Center, University of Chicago Medicine, USA; 2 Division of Gastroenterology, University of California [UC] San Diego and UC San Diego Health System, USA; 3 Division of Gastroenterology, Perelman School of Medicine of the University of Pennsylvania, USA; 4 Toronto Digestive Disease Associates, Canada; 5 Department of Pathology and Laboratory Medicine, Mt Sinai Hospital, Canada; 6 Salix Pharmaceuticals, USA

**David T. Rubin,^a^ Russell D. Cohen,^a^ William J. Sandborn,^b^ Gary R. Lichtenstein,^c^ Jeffrey Axler,^d^ Robert H. Riddell,^e^ Cindy Zhu,^f^ Andrew C. Barrett,^f^ Enoch Bortey,^f^ William P. Forbes^f^**

^a^Inflammatory Bowel Disease Center, University of Chicago Medicine, Chicago, IL, USA ^b^Division of Gastroenterology, University of California [UC] San Diego and UC San Diego Health System, San Diego, CA, USA ^c^Division of Gastroenterology, Perelman School of Medicine of the University of Pennsylvania, Philadelphia, PA, USA ^d^Toronto Digestive Disease Associates, Toronto, ON, Canada ^e^Department of Pathology and Laboratory Medicine, Mt Sinai Hospital, Toronto, ON, Canada ^f^Salix Pharmaceuticals, Raleigh, NC, USA

doi:10.1093/ecco-jcc/jjx032

This article has been updated to correct an error to the *n* value for patients randomised to treatment in Figure 1.

