# Supplementary material for: Budesonide Multimatrix Is Efficacious for Mesalamine-refractory, Mild to Moderate Ulcerative Colitis: A Randomised, Placebo-controlled Trial
Source: J Crohns Colitis. 2017 Mar 4;11(7):785–91. doi: 10.1093/ecco-jcc/jjx032 (PMC5881669; doi:10.1093/ecco-jcc/jjx032)

Supplementary Data for "Budesonide Multi-Matrix Is Efficacious for Mesalamine-Refractory, Mild to Moderate Ulcerative Colitis: A Randomized, Placebo-Controlled Trial"

David T. Rubin, Russell D. Cohen, William J. Sandborn, Gary R. Lichtenstein, Jeffrey Axler, Robert H. Riddell, Cindy Zhu, Andrew C. Barrett, Enoch Bortey, William P. Forbes

Supplementary Table 1. Biomarker concentrations [mITT population]

Assessment time	Budesonide MMX [*n* = 230]	Placebo [*n* = 228]	*P* value[a]
Serum C-reactive protein, mg/L, mean [SD]			
Baseline	8.4 [11.3]	9.2 [13.1]	
Week 8	8.6 [16.9]	6.4 [8.7][b]	0.67
Fecal calprotectin, mg/kg, mean [SD]			
Baseline	525.1 [459.6]	594.0 [869.6]	
Week 8	379.8 [362.2]	431.8 [382.7]	0.26

mITT, modified intent-to-treat; SD, standard deviation.
[a]*P* value for the difference in change from baseline between groups.
[b]Data available for 227 patients.

Supplementary Table 2. IBD-QOL scores [mITT population]

Assessment time	Budesonide MMX [*n* = 230]	Placebo [*n* = 228][a]	*P* value[b]
Total IBD-QOL score, mean [SD]			
Baseline	132.8 [31.4]	134.1 [32.5]	
Week 2	157.1 [31.6]	155.7 [32.6]	0.32
Week 4	164.6 [34.4]	160.2 [35.1]	0.04
Week 8	163.9 [39.4]	165.8 [36.8]	0.88
IBD-QOL bowel function subscale score, mean [SD]			
Baseline	40.5 [9.5]	40.5 [9.7]	
Week 2	49.2 [10.3][b]	48.0 [10.1]	0.2
Week 4	52.4 [11.5]	50.0 [10.9]	0.02
Week 8	52.2 [13.2]	52.2 [11.8]	1.0
IBD-QOL emotional function subscale score, mean [SD]			
Baseline	49.9 [13.5]	50.7 [14.1]	
Week 2	57.9 [13.5][c]	58.5 [13.7]	0.54
Week 4	60.3 [13.9]	59.7 [14.6]	0.13
Week 8	59.8 [15.6]	61.4 [14.8]	0.54
IBD-QOL systemic symptoms subscale score, mean [SD]			
Baseline	20.2 [5.3]	20.1 [5.7]	
Week 2	23.6 [5.1][c]	22.9 [5.6]	0.24
Week 4	24.3 [5.5]	23.7 [5.8]	0.28
Week 8	24.3 [6.1]	24.6 [5.9]	0.3
IBD-QOL social function subscale score, mean [SD]			
Baseline	22.2 [7.1]	22.8 [7.5]	
Week 2	26.3 [6.5][c]	26.2 [7.1]	0.2
Week 4	27.6 [6.6]	26.8 [7.4]	0.01
Week 8	27.6 [7.4]	27.6 [7.3]	0.16

[a]For total score and each subscale score, data available from 226 patients at baseline and week 2, and from 227 patients at weeks 4 and 8.

[b]*P* value for difference between groups in change from baseline.

[c]Data available for 228 patients.

IBD-QOL = inflammatory bowel disease quality of life; mITT = modified intent-to-treat population; SD = standard deviation.

Supplementary Table 3. Morning cortisol levels [safety population]

	Budesonide MMX [*n* = 255]		Placebo [*n* = 255]	
Assessment time	Mean [SD], µg/dL	Median, µg/dL	Mean [SD], µg/dL	Median, µg/dL
Baseline	12.5 [4.8]	12.0	12.0 [4.9]	11.0
Week 2	7.3 [5.2]	7.0	11.8 [4.5]	11.0
Week 4	7.3 [5.4]	7.0	12.4 [4.5]	12.0
Week 8	8.8 [6.5]	8.0	12.7 [4.6]	12.5

Supplementary Figure 1. Study design

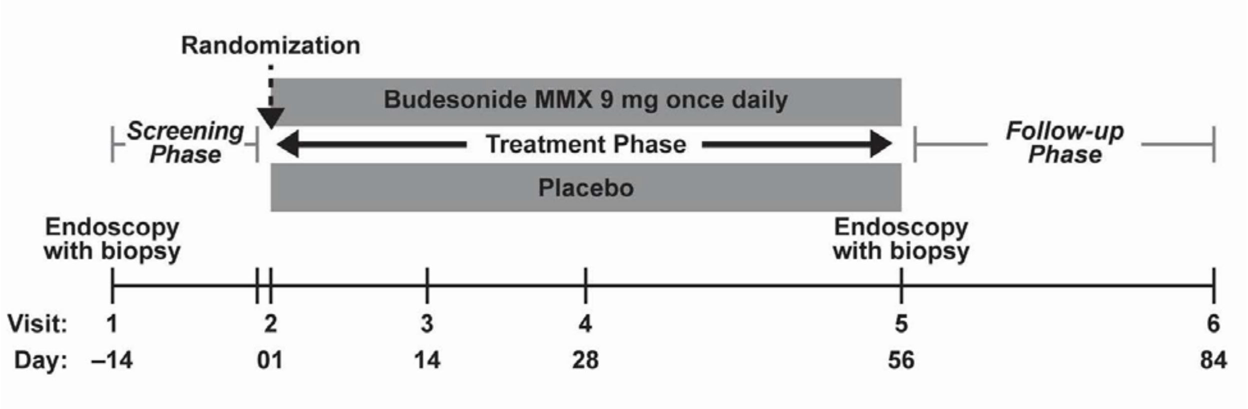

Supplement: Supplementary Material [file jjx032_suppl_supplementary_material.pdf]
